# Artificial Neural Network Approach for Assessing Mechanical Properties and Impact Performance of Natural-Fiber Composites Exposed to UV Radiation

**DOI:** 10.3390/polym16040538

**Published:** 2024-02-17

**Authors:** Khaled Nasri, Lotfi Toubal

**Affiliations:** Mechanical Engineering Department, The Innovation Institute in Eco Materials, Eco Products and Eco Energy (I2E3), Université du Quebec à Trois-Rivières (UQTR), C.P. 500, Trois-Rivières, QC G9A 5H7, Canada; khaled.nasri@uqtr.ca

**Keywords:** biocomposites, accelerated weathering, low-velocity impact response, ANN prediction

## Abstract

Amidst escalating environmental concerns, short natural-fiber thermoplastic (SNFT) biocomposites have emerged as sustainable materials for the eco-friendly production of mechanical components. However, their limited durability has prompted research into the experimental evaluation of the deterioration of the mechanical characteristics of SNFT biocomposites, particularly under the influence of ultraviolet rays. However, conducting tests to evaluate the mechanical properties can be time-consuming and expensive. In this study, an artificial neural network (ANN) model was employed to predict the mechanical properties (tensile strength) and the impact performance (resistance and absorbed energy) of polypropylene reinforced with 30 wt.% short flax or wood pine fibers (referred to as PP30-F or PP30-P, respectively). Eight parameters were collected from experimental studies. The ANN input parameters comprised nondestructive test results, including mass, hardness, roughness, and natural frequencies, while the output parameters were the tensile strength, the maximum impact load, and absorbed energy. The model was developed using the ANN toolbox in MATLAB. The linear coefficient of correlation and mean squared error were selected as the metrics for evaluating the performance function and accuracy of the ANN model. They calculate the relationship and the average squared difference between the predicted and actual values. The data analysis conducted by the models demonstrated exceptional predictive capability, achieving an accuracy rate exceeding 96%, which was deemed satisfactory. For both the PP30-F and PP30-P biocomposites, the ANN predictions deviated from the experimental data by 3, 5, and 6% with regard to the impact load, absorbed energy, and tensile strength, respectively.

## 1. Introduction 

Over the past two decades, biocomposites of short natural-fiber thermoplastics (SNFTs) obtained via injection molding have garnered considerable attention [1,2]. They are widely used in various industries, including construction, automotive, and packaging [3,4]. SNFT biocomposites offer high-specific mechanical properties and are eco-friendly [5,6,7,8]. Nevertheless, one major challenge in developing natural-fiber composites is their response to environmental factors, such as moisture, ultraviolet (UV) radiation, and heat, which can degrade their mechanical performance and limit their applicability, especially for outdoor applications [9,10,11,12,13,14,15]. 

Researchers have extensively investigated the aging of SNFTs via exposure to real climates (natural aging) [16,17,18,19] or simulated conditions in laboratory chambers (artificial aging) [20,21,22]. When SNFTs are exposed to UV rays and/or high temperatures, photo-oxidation reactions occur in the lignin of natural fibers, and moisture accelerates these reactions [10,23]. The velocity of photo-oxidation reactions in biocomposites depends on the chemical composition of the natural fibers. Peng et al. [22] investigated the impact of the chemical composition of natural fibers on the performance degradation of polypropylene reinforced with short wood fibers. They revealed that the lignin in natural fibers can act as a UV absorber, mitigating the degradation of the mechanical properties. Similarly, Nasri et al. [10] reported that biocomposites reinforced with wood pine fibers, which have high lignin content, exhibited less significant degradation in mechanical properties than flax fibers. For fibers, UV exposure initiates aging by causing absorption in the lignin structure, forming chromophore groups, like carboxylic acids, quinones, and hydroperoxy radicals, resulting in discoloration [13]. Polymers undergo photodegradation, leading to chain scission. This degradation in polymer matrices results in the formation of surface cracks, significantly reducing the tensile strength of biocomposites. Comprehensive reviews on the degradation mechanisms of biocomposites under UV exposure are available in [24,25,26]. Additionally, the mechanical properties of outdoor biocomposite structures deteriorate significantly more in a humid environment than in a dry one due to increased chemical reactivity [23]. While most studies on the impact of environmental factors on composites focus on the loss of mechanical properties using quasi-static tests, such as traction and flexion [23,24,25], it is important to note that these structures also experience low-speed impacts. Unfortunately, few studies have explored the behavior of biocomposites in response to such impacts [7].

Despite numerous and diverse research papers on natural-fiber-reinforced composites, the range of applications involving this type of material in engineering design is still limited. Notably, biocomposite structures designed for outdoor applications are vulnerable to low-speed impacts, and the strength of these materials becomes even more critical when exposed to the combined effects of UV radiation and/or moisture. It is well known that aging tests require numerous samples and are time-consuming and expensive. Aging (natural or artificial) requires exposure to various environmental conditions for months or even years [18,19]. Therefore, developing efficient prediction models to assess the mechanical resistance of biocomposites and their evolution based on exposure time to ultraviolet radiation and/or moisture is of great importance. It is crucial to acknowledge that laboratory-aging conditions may not perfectly replicate all environmental conditions encountered in practice. Aging in environmental chambers involves exposure to temperatures and light intensities higher than normal, as well as shorter wavelengths of light than in natural conditions. Additionally, natural aging is subject to unpredictable fluctuations in temperature, humidity, and other environmental factors, which can influence material degradation in complex and unpredictable ways. However, the advantage of accelerated aging tests lies in their ability to replicate, in weeks, the degradation of biocomposites that typically occurs outdoors over months or even years.

Artificial neural networks (ANNs) have emerged as highly effective methods for linear and nonlinear predictions concerning the mechanical properties of composite materials, considering their various constituents (such as fibers, matrix, and particles). An ANN creates predictive regression models based on experimental data [27]. Numerous researchers have successfully utilized ANN algorithms to predict the mechanical properties of biocomposite materials. For instance, Stamopoulos et al. [28] developed two ANN models trained using a multiscale methodology to predict the mechanical properties of matrix-dominated composites (for example, transverse strength, transverse stiffness, bending strength, flexural modulus, and short-beam strength), demonstrating consistency with experimental results. Yang et al. [29] developed an ANN model to predict the residual strength of carbon fiber-reinforced carbon (CFRC) following low-velocity impacts. The model, which was trained using finite-element analysis results, accurately established a nonlinear relationship between the impact parameters and residual strength, thus reducing computational costs and time compared to traditional methods. Fan et al. [30] trained an ANN-based model using limited test data to predict the tensile strengths of composite laminates with open holes. Altabey and Noori [31] developed a neural network model for predicting the fatigue life of CFRC, considering factors such as fatigue stress ratio, fiber orientation, materials, and loading conditions. Mohsin et al. [32] developed prediction models using ANN algorithms to predict the compressive strength and dry thermal conductivity of hemp-based biocomposites. Experimental records were used to train the models and demonstrate their accuracy and feasibility. These models offered significant time savings compared with laboratory tests. Zhang et al. [27] provided an in-depth overview of the use of ANNs for the mechanical modeling of composite materials. 

According to the literature, the use of ANN algorithms represents a robust approach for modeling complex nonlinear connections between inputs and outputs when obtaining a precise analytical expression, which is challenging. The strength of ANNs lies in their ability to analyze complex data, identify patterns and relationships, and accurately predict the mechanical behavior of biocomposites under various conditions. However, models should be based on easily measurable physical indicators. The data obtained from cut specimens of large components are primarily limited to laboratory use and are not applicable for in-service detection. Ideally, these data should be collected directly from a real structure without causing its destruction or alteration of its functionality. To achieve this, we have developed an efficient ANN-based model for predicting the low-velocity impact properties of aged biocomposites, specifically polypropylene reinforced with short flax or pine fibers. The novelty of our approach lies in predicting both mechanical and low-velocity impact properties through nondestructive testing, which involves measuring parameters such as mass, hardness, roughness, and resonant frequencies, providing the ANN model with its originality. By better understanding the behavior of these materials, it becomes possible to integrate them more effectively into various applications, thereby contributing to the development of environmentally friendly design solutions. In this context, tensile and impact samples were exposed to two accelerated aging programs: UV aging in dry and humid environments. Subsequently, we evaluated the changes in the properties of the biocomposites, and the proposed ANN model was finally validated with experimental results.

## 2. Methodology 

**Figure 1:**
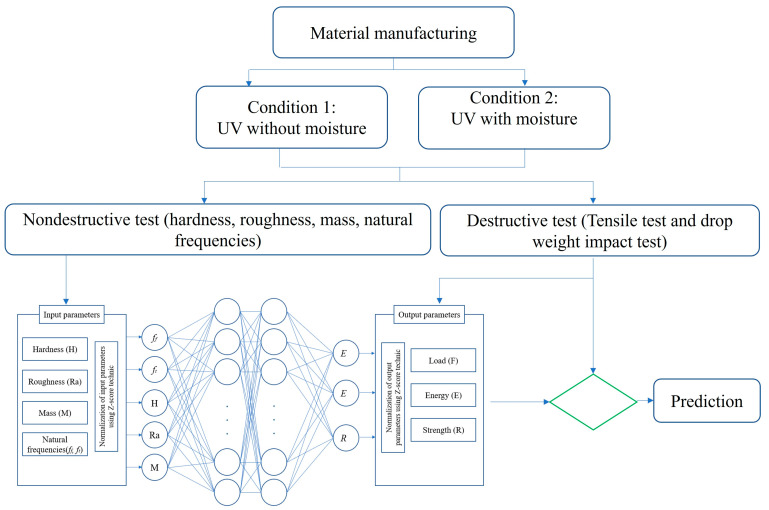
Prediction methodology.

This section summarizes the approach adopted to predict the long-term mechanical properties and low-velocity impact properties of the PP30-F and PP30-P biocomposites subjected to accelerated weathering. The collected data were used to develop an ANN model. Two aging conditions were used in this study: aging by UV rays with or without moisture. The input parameters of the model were the mass (M), hardness (H), mean roughness (Ra), and natural frequencies (bending and torsion modes, respectively, *f*_b_ and *f*_t_), and the output parameters were the maximum impact load and absorbed energy. After the ANN model was validated, such models were used to predict the tensile strength (R) and the impact performance (F and E). Figure 1 presents the methodology of this study.

### Artificial Neural Network 

**Figure 2:**
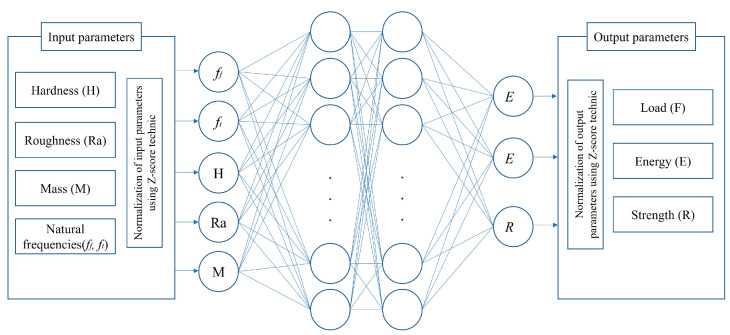
Architecture of the artificial neural network (ANN) algorithm.

An ANN is a network composed of perceptron cells linked by weighted interactions. Figure 2 presents the architecture of the ANN used in this study, which was divided into three parts: input, hidden, and output layers. The model was developed using the ANN toolbox implemented in MATLAB R2020 software. Before the ANNs were trained, the data were normalized to the range of −1 to +1 using the Z-score technique. This was done to ensure consistency with the transfer function used in the hidden and output layers. Then, the ANN models were trained, tested, and validated using a backpropagation algorithm. Subsequently, a set of data (input data and the corresponding output values) was applied to the developed ANN model to calibrate the weighting factors. A test set was used to select the best ANN through the calculation of the linear correlation coefficient (R^2^) and mean squared error (MSE).

A database was constructed on the basis of the experimental results. It included 70 sets of data used to validate the ANN model for predicting the impact performance (maximum load, absorbed energy, and tensile strength, that is, F, E, and R, respectively) of the biocomposites. Initially, six input parameters were selected: the hardness (H), mean roughness (Ra), and natural frequencies (torsion and bending modal, i.e., *f_t_* and *f_b_*, respectively).

## 3. Experimentation

### 3.1. Materials and Manufacturing 

The materials used in this study were biocomposites of polypropylene reinforced with either 30 wt.% flax fiber (FF30P233-00) or 30 wt.% pine wood fiber (WP30P233-00) purchased from Rhetech Inc. (Whitmore Lake, MI, USA). Flax fibers differ from pine fibers with regard to their chemical and geometric compositions. Flax fibers are rich in cellulose and have a higher length-to-diameter (L/d) ratio than pine fibers [10], whereas pine fibers are richer in lignin than flax fibers. This difference in fiber composition may influence the mechanical and aging properties of the studied short-fiber composites.

A 100-ton press (Zhafir Zeres series ZE900/210, Haitian Inc., Ningbo, China) was employed to perform injection molding of the impact samples in accordance with the ASTM D6226-21 standard [32]. The injection temperature was maintained at 200 °C. To prevent the occurrence of microvoids and porosity in the samples post-injection, the biocomposite granules were dried at 80 °C for 2 h prior to injection.

### 3.2. Aging Conditions 

Two environmental conditions were considered in this study:

- Condition 1 (UV without moisture): The samples were subjected to UV aging using UVA-340 fluorescent lamps emitting irradiance at a wavelength of 340 nm. Aging was performed using a QUV/SE aging apparatus (Q-Lab Co., Westlake, OH, USA). Over a period of two months, the samples were exposed to 8 h of UV radiation each day at an irradiance of 1.55 W/m^2^, and the temperature was maintained at 60 °C.
- Condition 2 (UV with moisture): The samples were subjected to the same conditions as Condition 1 for two months. However, after each UV exposure at 60 °C, the samples were subjected to 4 h of water condensation at 50 °C.

The aging conditions were conducted in accordance with ASTM G154-23 [33], the standard practice for artificial UV-aging of non-metallic materials.

### 3.3. Experimental Setup and Procedure

#### 3.3.1. Mass Measurement

Specimen mass was measured using a precision electronic scale with accuracy up to 10^−3^ g, providing the mass (M) in kilograms (kg).

#### 3.3.2. Roughness Measurement

Surface roughness was evaluated via a 3D laser confocal microscope (Keyence, Japan). The roughness parameter Ra was determined in millimeters (mm).

#### 3.3.3. IET (Impulse Excitation Technic)

Impact Echo Testing (IET) was conducted following ASTM E-1876-09 [34] on an IMCE machine. Signal processing, facilitated by Resonant Frequency and Damping Analyzer (RFDA) Professional software, yielded resonance frequency (f) results in Hertz (Hz).

#### 3.3.4. Hardness Test

Hardness assessments adhered to ASTM D785-08 [35] standards utilizing an HM-100 Economical manual-type hardness testing machine (810-124). The resulting hardness values (H) are reported in HRC (Rockwell C scale).

#### 3.3.5. Tensile Test

We utilized an Instron model LM-U150 electromechanical testing apparatus, which was equipped with a 10 KN load cell, to rigorously examine the tensile strength properties of materials. Our experimental protocol adhered to the ASTM D638 standard for such tests. The tests were performed at a controlled displacement rate of 1 mm/min.

#### 3.3.6. Drop-Weight Impact Test

The ASTM D-5628 [36] standard guided drop-weight impact tests on an Instron machine (Model CEAST 9350) equipped with a 22 kN load cell. Employing an initial impact energy of 5 Joules and a 5.4 kg impactor, impact force (F) values were measured in kilogram meters per second squared (kg⋅m⋅s^−2^), while impact energy (E) values were quantified in Joules (J).

**Figure A1:**
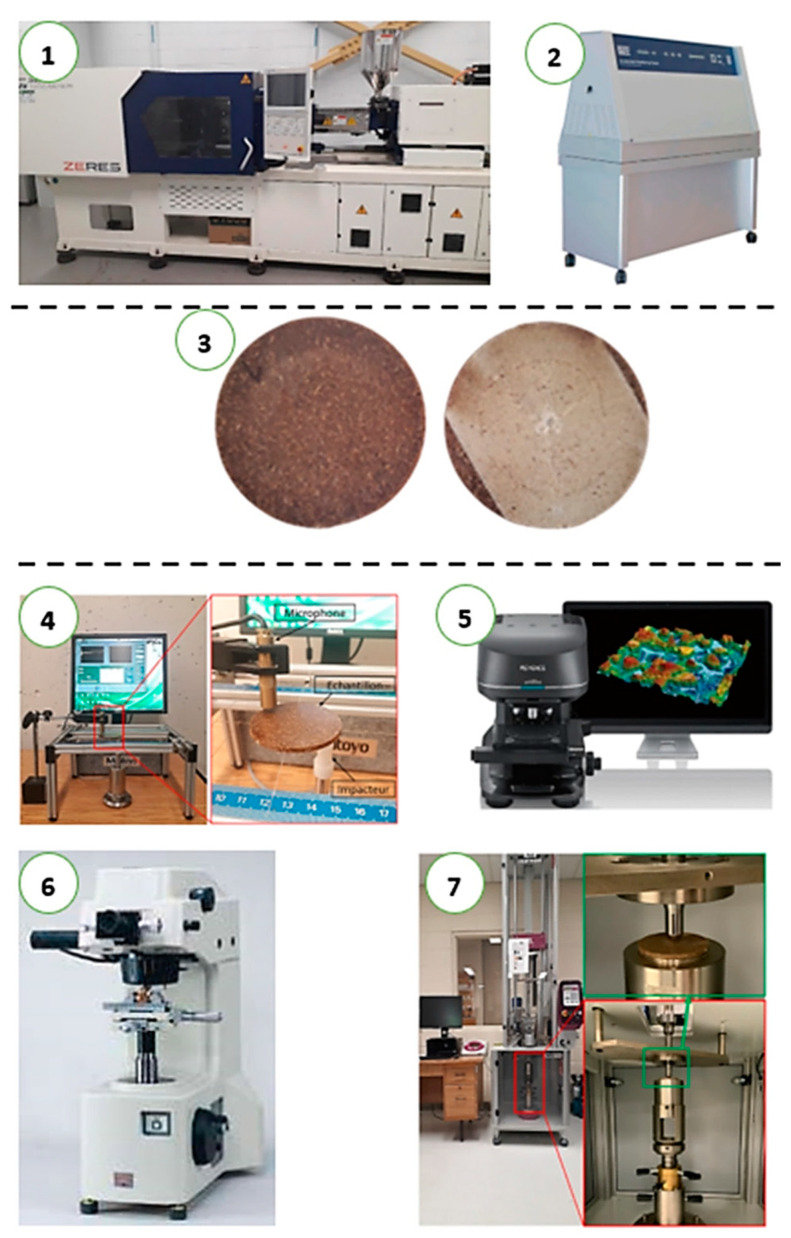
Equipment and samples used: 1—injection molding machine; 2—accelerated-weathering machine; 3—examples of unaged and aged PP30-F samples; 4—IET device; 5—3D confocal microscopy device; 6—hardness test machine; 7—drop-weight impact test machine.

The details of each test (device and measurement method) are presented in Appendix A (Table A1). The equipment and samples used in this study are presented in Appendix B (Figure A1).

## 4. Results and Discussion

### 4.1. Experimental Results

The evaluation results are presented in Table 1. Both biocomposites exhibited changes in their physical and mechanical properties over time. These changes were more significant under the second treatment (UV irradiation with moisture), possibly because photo-oxidation reactions are the main contributors to SNFT property degradation [10], and moisture accelerates these reactions [23]. Consequently, a higher degree of degradation was observed under the second condition (UV irradiation with moisture) for both materials.

#### 4.1.1. Mass

The masses of the samples decreased with an increase in the exposure time for both conditions (UV with and without moisture). These mass losses were mainly due to the degradation of natural fibers by photo-oxidation reactions [23].

#### 4.1.2. Hardness 

**Figure 3:**
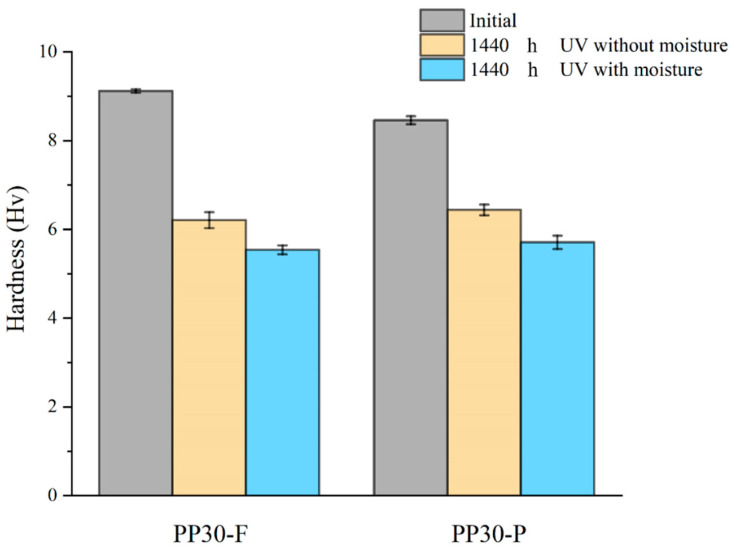
Average hardness evolution of PP30-F and PP30-P biocomposites as function of exposure time.

Prior to aging, the PP30-F biocomposites exhibited higher hardness than the PP30-P biocomposites; the corresponding hardness values were H = 9.12 and 8.72 HRC, respectively. After aging, a reduction in the hardness was observed for both materials, as shown in Figure 3. However, after 1440 h of UV exposure under dry conditions, the measured hardness of the PP30-F biocomposite was 6.21 HRC, while that of the PP30-P biocomposite was 6.44 HRC. Similarly, under humid conditions, the corresponding values were 5.54 and 5.71 HRC, respectively. This reduction is mainly attributed to the scission of the polymer chains, which led to the formation of surface cracks and embrittlement of the material. The number of chain scissions increased with the exposure time, resulting in shorter polymer chains and degradation of all the mechanical properties. PP30-P exhibited a smaller hardness loss than PP30-F, which can be explained by the antioxidant effect of the lignin in pine fibers [29].

#### 4.1.3. Roughness 

**Figure 4:**
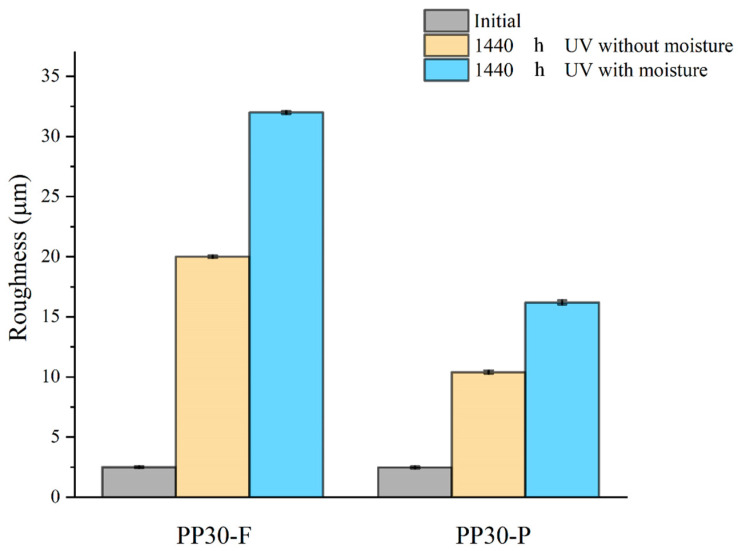
Average roughness of unaged and aged PP30-F and PP30-P biocomposites after 1440 h of exposure.

The measured results for the surface roughness presented in Figure 4 indicate that both biocomposites initially had smooth and intact surfaces with a roughness of approximately 2.5 μm. However, after UV exposure in dry or moist conditions, both biocomposites exhibited rough surfaces. The increase in the surface roughness of the UV-exposed biocomposites is attributed to polymer-chain scission resulting from photo-oxidation. Polymer-chain scission is also responsible for the formation of microcracks on the surface of SNFT biocomposites during aging [23]. The PP30-P biocomposite exhibited fewer large cracks than the PP30-F biocomposite under both conditions. The lignin content of pinewood fibers is at least seven times higher than that of flax fibers. This suggests that the presence of lignin in pinewood had an antioxidant effect, delaying surface degradation, as previously reported [10]. After 1440 h of UV exposure in dry conditions, the average roughness (Ra) measured for the PP30-F biocomposite was 19.86 µm, and that for the PP30-P biocomposite was 10.02 µm. Similarly, the corresponding values for humid conditions were 32.41 and 16.73 µm, respectively.

#### 4.1.4. Natural Frequencies 

**Figure 5:**
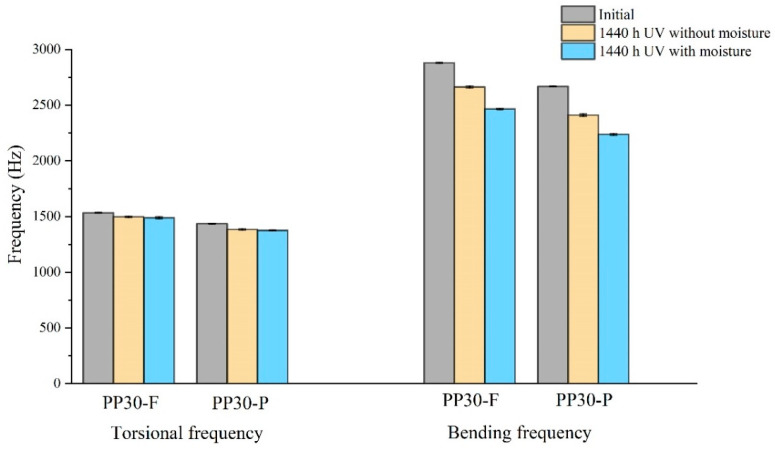
Bending and torsional frequencies of unaged and aged PP30-F and PP30-P biocomposites after 1440 h of exposure.

Figure 5 shows the natural frequencies of the unaged and aged biocomposite samples at 1440 h. The results indicate reductions in the natural frequencies. The UV irradiation reduced the resonant frequencies of the composites by degrading the polymer materials in both the matrix and the reinforcing fibers. In the case of UV aging combined with moisture, the resonant-frequency reduction was accelerated. Photo-oxidation causes the splitting of the polymer chains of the thermoplastic matrix, resulting in surface microcracks. When composites are exposed to moisture, water can be absorbed by natural fibers or by the interfaces between the fibers and matrix. This water absorption causes swelling of the fibers and matrix, which affects the fiber–matrix adhesion and the mechanical properties of the composite, reducing the resonant frequency.

#### 4.1.5. Tensile Test 

**Figure 6:**
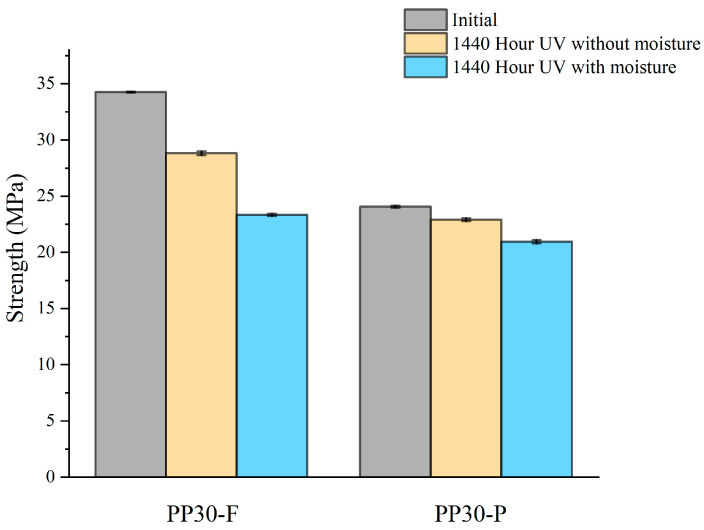
Strength of unaged and aged PP30-F and PP30-P biocomposites after 1440 h of exposure.

Figure 6 shows the tensile strength of both aged and unaged biocomposite samples after 1440 hours. The results reveal a decrease in the tensile strength following aging. This reduction in tensile strength is attributed to the effects of UV radiation on the polymeric materials within the natural fibers, particularly lignin, and the polypropylene matrix. This may be explained by the fact that photo-oxidation induces superficial microcracks in the biocomposites, which act as stress concentration points within the samples, consequently leading to a degradation in mechanical properties such as tensile strength. When UV exposure is combined with moisture, the degradation process is amplified. Indeed, moisture accelerates the photo-oxidation reactions, further promoting degradation.

#### 4.1.6. Low-Velocity Impact Properties 

**Figure 7:**
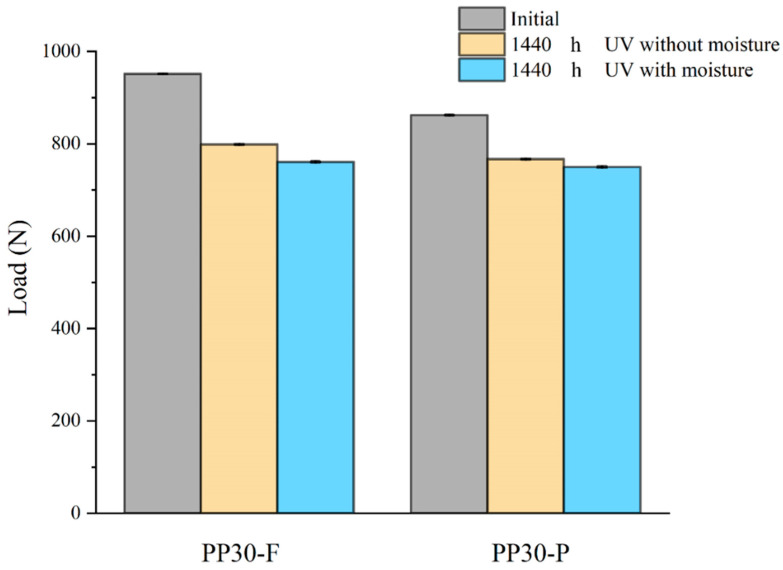
Maximum impact loads of unaged and aged PP30-F and PP30-P biocomposites after 1440 h of exposure.

**Figure 8:**
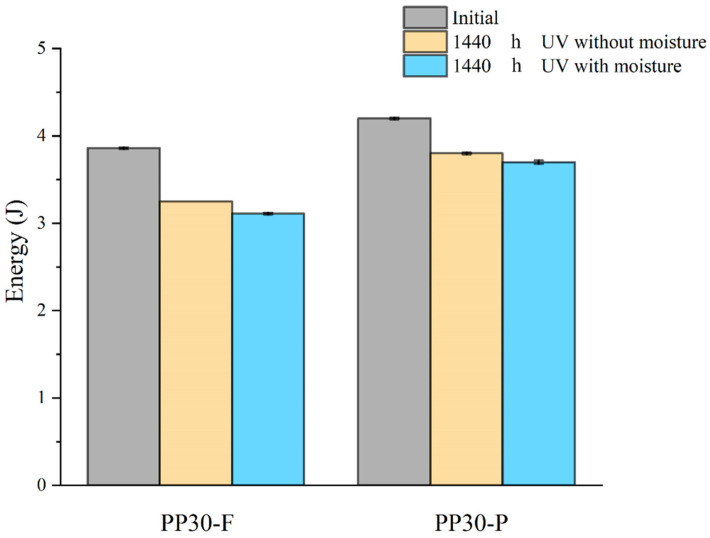
Absorbed energy evolution of PP30-F and PP30-P biocomposites as function of exposure time.

The low-velocity impact properties, such as the impact resistance and absorbed energy of aged biocomposites, were studied to evaluate the effects of aging on their behavior under impact loads. The results (Figure 7 and Figure 8) indicated significant changes in these properties with an increase in the exposure time, which is mainly attributed to the formation of microsurface cracks during the aging of the biocomposite samples. These microcracks acted as initiation points for damage under an impact load, amplifying the local stresses within the biocomposites. The amplification of local stresses resulting from surface microcracks reduced the tensile strength and absorbed energy of the aged biocomposites.

The PP30-P biocomposites exhibited less degradation than the PP30-F biocomposites with regard to their impact properties. This difference is attributed to the antioxidant effect of the lignin present in wood pine fibers. Lignin suppresses crack propagation during impact by delaying the initiation and evolution of surface microcracks.

Prior to aging, the PP30-F biocomposites exhibited higher strength than the PP30-P biocomposites, with a maximum load (F) of 951.72 N and energy absorption (E) of 3.86 J, compared with a maximum load of 862.28 N and energy absorption of 4.21 J for PP30-P. However, after aging, the PP30-F biocomposites exhibited more significant degradation in both properties. After 1440 h of exposure, the PP30-F biocomposites exhibited a maximum load of 798.84 N and energy absorption of 3.25 J in the first condition (UV without moisture) and a maximum load of 867.18 N and energy absorption of 3.78 J in the second condition (UV with moisture). Meanwhile, the PP30-P biocomposites exhibited a maximum load of 760.82 N and energy absorption of 3.11 J in the first condition and a maximum load of 749.94 N and energy absorption of 3.68 J in the second condition.

Table 1 presents the experimental results for the physical and mechanical properties.

### 4.2. ANN Approach 

#### 4.2.1. ANN Model Validation 

**Figure 9:**
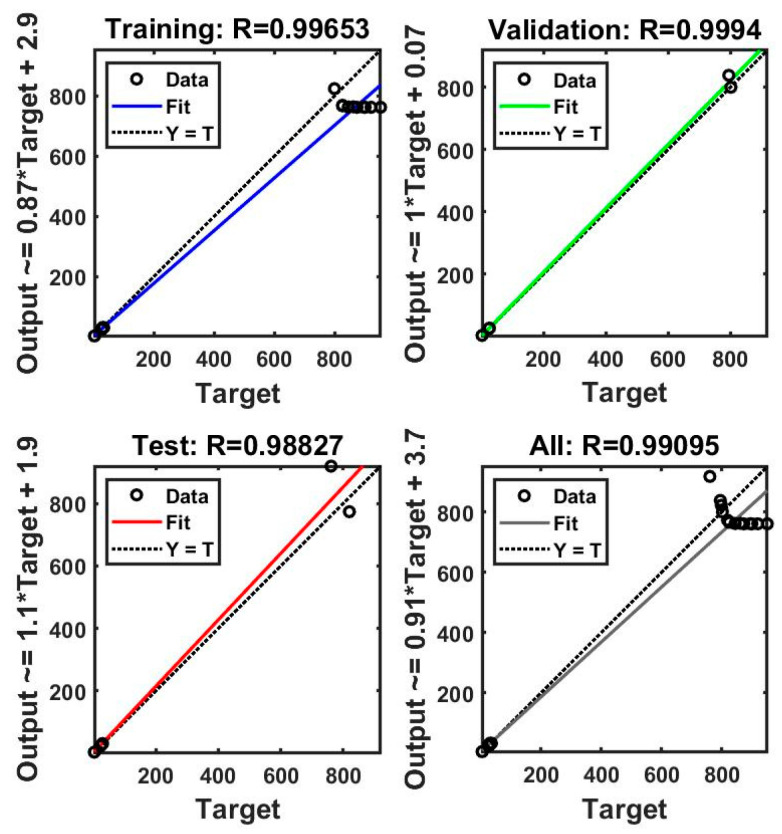
Linear coefficient regression of the artificial neural network (ANN) model (PP30-F).

**Figure 10:**
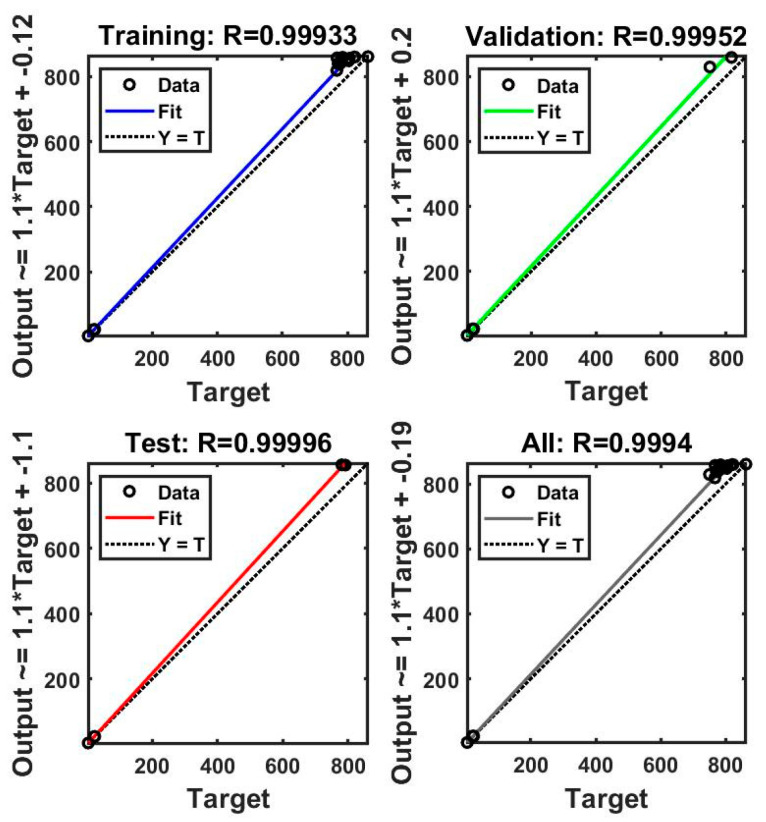
Linear coefficient regression of the artificial neural network (ANN) model (PP30-P).

The performance of the ANN model was evaluated according to the convergence of the MSE. The best validation performance was observed after four epochs (MSE = 22). Figure 9 and Figure 10 present plots of the linear regression coefficients. As shown, the model fit the data well; the global correlation coefficients (R) in the case of PP30-F were 0.999 for training, 0.997 for testing, and 0.999 for validation, and in the case of PP30-P, they were 0.997, 0.999, and 0.999, respectively. In addition, the training, testing, and validation stages of the model for prediction were positive. This suggests that the model learned effectively, generalized well to new data, and did not overfit the training dataset. It indicates a promising performance and adds credibility to the model’s ability to make accurate predictions.

#### 4.2.2. ANN Prediction 

**Figure 11:**
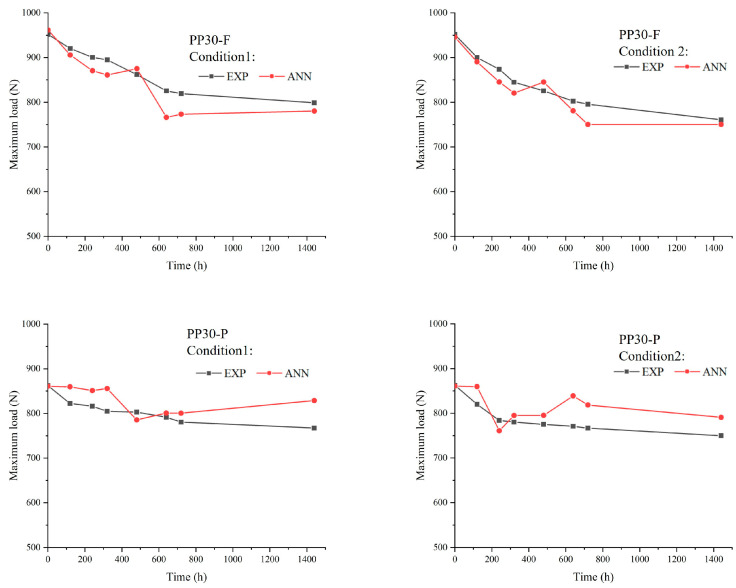
Comparison between experimental and artificial neural network (ANN) results for the impact load.

**Figure 12:**
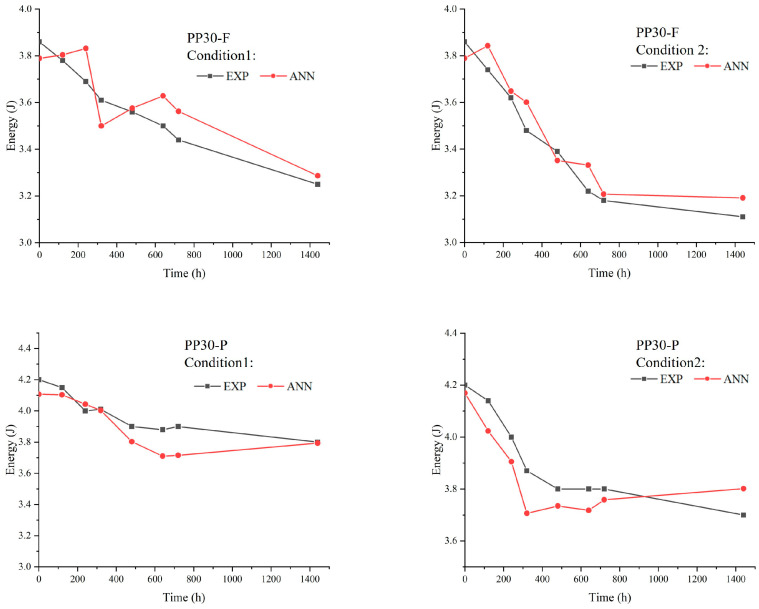
Comparison between experimental and artificial neural network (ANN) results for the absorbed energy.

**Figure 13:**
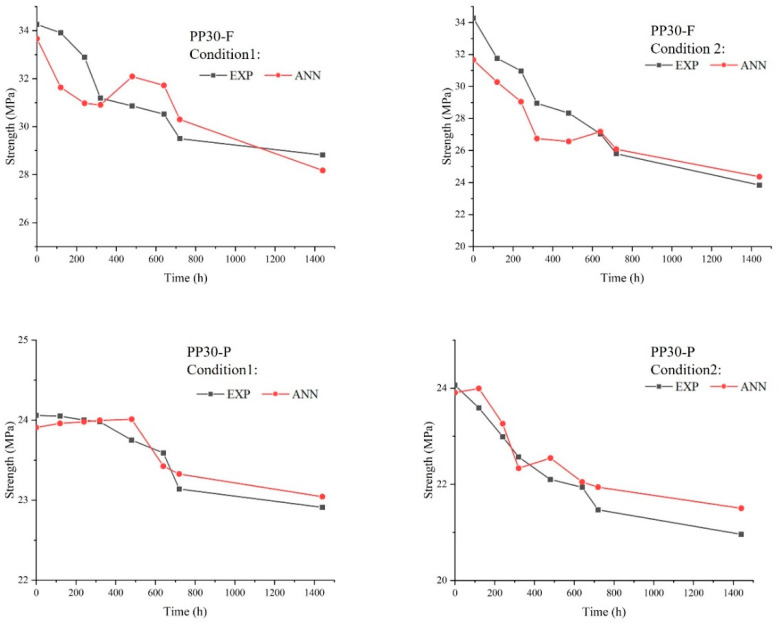
Comparison between experimental and artificial neural network (ANN) results for the tensile strength.

Figure 11, Figure 12 and Figure 13 present comparisons between the prediction results of the ANN model and the experimentally obtained results. Overall, the proposed model provided accurate results. The ANN results agreed well with the experimental data concerning the impact load, energy absorbed, and tensile strength, with maximum errors of 3, 5%, and 6%, respectively. However, it is important to recognize that despite the overall accuracy of our predictions, slight differences between the results of the ANN algorithm and the experimental data may appear. These deviations could arise from several factors, such as natural variations in material properties, environmental conditions during experimental testing, or even potential inaccuracies in the parameters of the algorithm itself. However, despite these minor deviations, our model is capable of accurately predicting the mechanical impact performance of aged SNFT biocomposites.

## 5. Conclusions

In external applications, biocomposites are vulnerable to environmental aging and low-velocity impacts. Aging reduces the resistance of biocomposites to low-impact collisions. Thus, to avoid damage, it is important to predict the impact resistance of aged biocomposites. In this study, a novel ANN prediction model was developed to predict the durability of aged biocomposites with regard to their mechanical and impact properties. This network was applied to two biocomposite materials (polypropylene reinforced with 30 wt.% flax or pine fibers) under two environmental conditions (UV aging in a dry or moist environment). Unaged and aged biocomposites were evaluated via nondestructive tests, such as mass, hardness, roughness, and IET tests, as well as destructive tests (tensile tests and low-velocity impact tests) to evaluate these properties with respect to the exposure time. The results indicated that all the mechanical properties were degraded after aging. The degradation was more significant under Condition 2 (UV with moisture) than under Condition 1 (UV without moisture). Moreover, PP30-F exhibited a higher degree of degradation than PP30-P under both conditions. Subsequently, the impact properties of the two composite materials (tensile strength and absorbed impact energy) were predicted using the developed ANN algorithm, for which nondestructive test results were used as inputs. The proposed ANN model proved to be a reliable prediction tool, as indicated by strong agreement between the experimental and predicted results. The correlation coefficient R was 0.999 for the two biocomposites.

## Figures and Tables

**Table 1:** Experimental results.

Mat	Condition	Time (h)	M (g)	H	Ra	*f*_t_	*f*_b_	F	E	R
				(HRC)	(mm)	(Hz)	(Hz)	(N)	(J)	(MPa)
PP30-F	UV without moisture	0	9.706 (0.01)	9.12 (0.12)	2.5 (0.01)	1534 (3.45)	2880 (9.51)	951.72 (1.12)	3.86 (0.01)	34.26 (0.01)
		120	9.706 (0.02)	8.87 (0.15)	2.8 (0.02)	1530 (10.12)	2887 (9.29)	920.17 (1.18)	3.78 (0.02)	33.91 (0.03)
		240	9.702 (0.01)	8.14 (0.21)	3.4 (0.02)	1531 (3.77)	2886 (4.37)	900.51 (1.28)	3.69 (0.02)	32.89 (0.02)
		320	9.701 (0.02)	7.92 (0.18)	6.6 (0.02)	1518 (7.48)	2815 (6.29)	894.74 (2.32)	3.61 (0.02)	31.19 (0.05)
		480	9.548 (0.04)	7.82 (0.09)	7.8 (0.01)	1502 (3.45)	2755 (7.48)	861.93 (1.78)	3.56 (0.03)	30.86 (0.03)
		640	9.341 (0.02)	7.48 (0.19)	9.4 (0.03)	1504 (6.48)	2743 (5.59)	825.46 (1.81)	3.50 (0.03)	30.52 (0.01)
		720	9.137 (0.01)	7.25 (0.21)	13 (0.02)	1499 (8.55)	2733 (7.88)	819.36 (1.89)	3.44 (0.03)	29.5 (0.01)
		1440	9.012 (0.01)	6.21 (0.22)	19.86 (0.02)	1498 (6.84)	2663 (6.79)	798.84 (2.22)	3.25 (0.02)	28.82 (0.06)
	UV with moisture	120	9.704 (0.01)	6.91 (0.15)	4.1 (0.02)	1532 (8.87)	2856 (7.59)	900.14 (1.56)	3.74 (0.01)	31.76 (0.04)
		240	9.698 (0.02)	6.74 (0.18)	6.4 (0.01)	1530 (7.13)	2764 (8.49)	873.82 (4.27)	3.62 (0.01)	30.97 (0.01)
		320	9.645 (0.04)	6.51 (0.19)	11.6 (0.03)	1501 (9.44)	2667 (8.71)	844.84 (3.48)	3.48 (0.01)	28.95 (0.01)
		480	9.412 (0.02)	6.45 (0.21)	12.5 (0.01)	1500 (3.19)	2565 (4.14)	825.51 (1.12)	3.39 (0.02)	28.34 (0.03)
		640	9.104 (0.04)	6.22 (0.11)	18 (0.01)	1495 (8.42)	2529 (8.46)	802.52 (1.29)	3.22 (0.02)	27.04 (0.02)
		720	9.016 (0.02)	6.01 (0.17)	26 (0.02)	1493 (8.42)	2486 (8.46)	795.59 (3.82)	3.18 (0.02)	25.8 (0.05)
		1440	8.802 (0.03)	5.54 (0.14)	32.41 (0.02)	1490 (9.43)	2466 (7.68)	760.82 (2.32)	3.11 (0.02)	23.84 (0.03)
PP30-P	UV without moisture	0	9.892 (0.03)	8.72 (0.18)	2.47 (0.03)	1436 (7.12)	2668 (6.41)	862.3 (3.72)	4.2 (0.01)	24.06 (0.02)
		120	9.870 (0.03)	8.45 (0.19)	3.00 (0.03)	1415 (9.28)	2610 (9.11)	822.15 (2.32)	4.15 (0.01)	24.05 (0.04)
		240	9.841 (0.02)	8.11 (0.27)	4.47 (0.03)	1416 (6.66)	2630 (9.34)	816.2 (4.32)	4 (0.02)	24.04 (0.03)
		320	9.832 (0.01)	7.85 (0.24)	5.56 (0.02)	1403 (9.65)	2560 (9.40)	804.74 (2.32)	4.01 (0.02)	24.031 (0.05)
		480	9.800 (0.02)	7.64 (0.19)	6.71 (0.02)	1388 (8.41)	2501 (3.54)	802.9 (2.42)	3.9 (0.01)	24.01 (0.01)
		640	9.394 (0.04)	7.45 (0.21)	7.91 (0.02)	1389 (8.37)	2490 (8.41)	791.44 (3.42)	3.88 (0.02)	24.04 (0.02)
		720	9.234 (0.02)	7.12 (0.17)	8.41 (0.01)	1385 (9.35)	2480 (8.19)	780.6 (1.77)	3.9 (0.03)	23.14 (0.03)
		1440	9.108 (0.03)	6.44 (0.19)	10.02 (0.01)	1384 (7.19)	2411 (8.38)	767.2 (2.82)	3.8 (0.02)	22.91 (0.04)
	UV with moisture	120	9.801 (0.02)	8.24 (0.22)	3.13 (0.03)	1417 (8.48)	2600 (10.21)	820.18 (1.68)	4.14 (0.01)	23.59 (0.02)
		240	9.851 (0.01)	7.81 (0.27)	3.27 (0.03)	1415 (6.61)	2510 (4.68)	783.9 (3.91)	4 (0.01)	22.99 (0.05)
		320	9.762 (0.02)	7.57 (0.29)	4.15 (0.03)	1387 (6.39)	2415 (5.79)	780.21 (3.10)	3.87 (0.01)	22.57 (0.02)
		480	9.571 (0.04)	6.94 (0.21)	5.16 (0.03)	1386 (7.55)	2315 (9.12)	775,00 (1.88)	3.8 (0.02)	22.1 (0.03)
		640	9.115 (0.02)	6.64 (0.12)	10.4 (0.02)	1381 (8.49)	2280 (9.11)	770.74 (2.94)	3.80 (0.01)	21.94 (0.06)
		720	9.104 (0.02)	6.21 (0.28)	12.5 (0.02)	1379 (7.22)	2238 (4.51)	766.8 (1.11)	3.8 (0.01)	21.47 (0.04)
		1440	9.011 (0.03)	5.71 (0.21)	16.73 (0.01)	1376 (6.75)	2238 (7.64)	749.9 (1.22)	3.7 (0.03)	20.96 (0.03)

Values in parentheses are standard deviations.

## Data Availability

Data are contained within the article.

## Appendix A

**Table A1:** Tests and characterization methods for the short natural-fiber thermoplastic (SNFT) biocomposites.

Test	Method	Machine	Properties
Physical properties			
Mass measurement	Measuring tape	Electronic scale Accuracy 10^–3^ g	M (kg)
Roughness measurement	Laser surface scan	3D laser confocal microscope (Keyence, Canada)	Ra (mm)
IET	ASTM E-1876-09	IMCE machine Signal-processing software: resonant frequency and damping analyzer (RFDA)	*f* (Hz)
Mechanical properties			
Hardness test	ASTM E329	810-124: HM-100 Economical manual type	H (HRC)
Drop-weight impact test	ASTM D-5628	Instron machine Model CEAST 9350, 22 kN load cell initial impact energy of 5 J, mass of impactor: 5.4 kg	F (kg⋅m⋅s^–2^), E (J)

## References

[1] Pickering K.L., Efendy M.G.A., Le T.M. (2016). A review of recent developments in natural fibre composites and their mechanical performance. Compos. Part A Appl. Sci. Manuf..

[2] Tanguy M., Bourmaud A., Beaugrand J., Gaudry T., Baley C. (2018). Polypropylene reinforcement with flax or jute fibre; Influence of microstructure and constituents properties on the performance of composite. Compos. Part B Eng..

[3] Nurazzi N.M., Asyraf M.R.M., Khalina A., Abdullah N., Aisyah H.A., Rafiqah S.A., Sabaruddin F.A., Kamarudin S.H., Norrrahim M.N.F., Ilyas R.A. (2021). A Review on Natural Fiber Reinforced Polymer Composite for Bullet Proof and Ballistic Applications. Polymers.

[4] Lau K.-T., Hung P.-Y., Zhu M.-H., Hui D. (2018). Properties of natural fibre composites for structural engineering applications. Compos. Part B Eng..

[5] Alonso-Montemayor F.J., Espinach F.X., Tarrés Q., Alcalà M., Delgado-Aguilar M., Mutjé P. (2023). The Evolution of the Intrinsic Flexural Strength of Jute Strands after a Progressive Delignification Process and Their Contribution to the Flexural Strength of PLA-Based Biocomposites. Polymers.

[6] Ferreira E.d.S.B., da Silva F.S., Luna C.B.B., Costa A.R.d.M., de Sousa F.M., de Carvalho L.H., Wellen R.M.R., Araújo E.M. (2024). Toward Producing Biopolyethylene/Babassu Fiber Biocomposites with Improved Mechanical and Thermomechanical Properties. Polymers.

[7] Nasri K., Loranger É., Toubal L. (2023). Effect of cellulose and lignin content on the mechanical properties and drop-weight impact damage of injection-molded polypropylene-flax and -pine fiber composites. J. Compos. Mater..

[8] Koffi A., Koffi D., Toubal L. (2020). Mechanical properties and drop-weight impact performance of injection-molded HDPE/birch fiber composites. Polym. Test..

[9] Stark N.M., Matuana L.M. (2003). Ultraviolet weathering of photostabilized wood-flour-filled high-density polyethylene composites. J. Appl. Polym. Sci..

[10] Nasri K., Toubal L., Loranger É., Koffi D. (2022). Influence of UV irradiation on mechanical properties and drop-weight impact performance of polypropylene biocomposites reinforced with short flax and pine fibers. Compos. Part C Open Access.

[11] Nasri K., Toubal L. (2023). Experimental and numerical investigation of damage and mechanical property retention by bio-composite plastic made with flax or pinewood fiber and aged by exposure to ultraviolet light. J. Build. Eng..

[12] Kallakas H., Ayansola G.S., Tumanov T., Goljandin D., Poltimäe T., Krumme A., Kers J. (2019). Influence of birch false heartwood on the physical and mechanical properties of wood-plastic composites. BioResources.

[13] Matuana L.M., Jin S., Stark N.M. (2011). Ultraviolet weathering of HDPE/wood-flour composites coextruded with a clear HDPE cap layer. Polym. Degrad. Stab..

[14] Liu T., Liu X., Feng P. (2020). A comprehensive review on mechanical properties of pultruded FRP composites subjected to long-term environmental effects. Compos. Part B Eng..

[15] Nagaraja S., Anand P.B., Naik R.N.M., Gunashekaran S. (2022). Effect of aging on the biopolymer composites: Mechanisms, modes and characterization. Polym. Compos..

[16] Fabiyi J.S., McDonald A.G., Wolcott M.P., Griffiths P.R. (2008). Wood plastic composites weathering: Visual appearance and chemical changes. Polym. Degrad. Stab..

[17] Thirmizir M.Z.A., Ishak Z.A.M., Taib R.M., Rahim S., Jani S.M. (2011). Natural Weathering of Kenaf Bast Fibre-Filled Poly(Butylene Succinate) Composites: Effect of Fibre Loading and Compatibiliser Addition. J. Polym. Environ..

[18] Belec L., Nguyen T., Nguyen D., Chailan J. (2015). Comparative effects of humid tropical weathering and artificial ageing on a model composite properties from nano- to macro-scale. Compos. Part A Appl. Sci. Manuf..

[19] Soccalingame L., Perrin D., Bénézet J.-C., Bergeret A. (2016). Reprocessing of UV-weathered wood flour reinforced polypropylene composites: Study of a natural outdoor exposure. Polym. Degrad. Stab..

[20] Badji C., Beigbeder J., Garay H., Bergeret A., Bénézet J.-C., Desauziers V. (2018). Exterior and under glass natural weathering of hemp fibers reinforced polypropylene biocomposites: Impact on mechanical, chemical, microstructural and visual aspect properties. Polym. Degrad. Stab..

[21] Badji C., Beigbeder J., Garay H., Bergeret A., Bénézet J.-C., Desauziers V. (2018). Correlation between artificial and natural weathering of hemp fibers reinforced polypropylene biocomposites. Polym. Degrad. Stab..

[22] Peng Y., Liu R., Cao J., Chen Y. (2014). Effects of UV weathering on surface properties of polypropylene composites reinforced with wood flour, lignin, and cellulose. Appl. Surf. Sci..

[23] Azwa Z., Yousif B., Manalo A., Karunasena W. (2013). A review on the degradability of polymeric composites based on natural fibres. Mater. Des..

[24] Chang B.P., Mohanty A.K., Misra M. (2020). Studies on durability of sustainable biobased composites: A review. RSC Adv..

[25] Dittenber D.B., GangaRao H.V. (2012). Critical review of recent publications on use of natural composites in infrastructure. Compos. Part A Appl. Sci. Manuf..

[26] Zhang Z., Friedrich K. (2003). Artificial neural networks applied to polymer composites: A review. Compos. Sci. Technol..

[27] Stamopoulos A., Tserpes K., Dentsoras A. (2018). Quality assessment of porous CFRP specimens using X-ray Computed Tomography data and Artificial Neural Networks. Compos. Struct..

[28] Yang B., Fu K., Lee J., Li Y. (2021). Artificial Neural Network (ANN)-Based Residual Strength Prediction of Carbon Fibre Reinforced Composites (CFRCs) After Impact. Appl. Compos. Mater..

[29] Fan H.-T., Wang H. (2014). Predicting the Open-Hole Tensile Strength of Composite Plates Based on Probabilistic Neural Network. Appl. Compos. Mater..

[30] Altabey W.A., Noori M. (2017). Fatigue life prediction for carbon fibre/epoxy laminate composites under spectrum loading using two different neural network architectures. Int. J. Sustain. Mater. Struct. Syst..

[31] Khan M.A., Aslam F., Javed M.F., Alabduljabbar H., Deifalla A.F. (2022). New prediction models for the compressive strength and dry-thermal conductivity of bio-composites using novel machine learning algorithms. J. Clean. Prod..

[32] Standard Test Method for Open Cell Content of Rigid Cellular Plastics.

[33] Standard Practice for Operating Fluorescent Ultraviolet (UV) Lamp Apparatus for Exposure of Materials.

[34] Standard Test Method for Dynamic Young’s Modulus, Shear Modulus, and Poisson’s Ratio by Impulse Excitation of Vibration.

[35] Standard Test Method for Rockwell Hardness of Plastics and Electrical Insulating Materials.

[36] Standard Test Method for Tensile Properties of Plastics.

